# Evaluation of Gas Explosion Injury Based on Analysis of Rat Serum Profile by Ultra-Performance Liquid Chromatography/Mass Spectrometry-Based Metabonomics Techniques

**DOI:** 10.1155/2020/8645869

**Published:** 2020-07-28

**Authors:** Xinwen Dong, Weidong Wu, Sanqiao Yao, Jia Cao, Ling He, Houcheng Ren, Wenjie Ren

**Affiliations:** ^1^Department of Environmental and Occupational Health, School of Public Health, Xinxiang Medical University, Xinxiang, Henan Province, China 453003; ^2^Institute of Toxicology, College of Preventive Medicine, Army Medical University, Chongqing, China 400038; ^3^Department of Ophthalmology, The Affiliated 371st Hospital, Xinxiang Medical University, Xinxiang, Henan Province, China 453000; ^4^Department of Human Resources, Sanquan College, Xinxiang Medical University, Xinxiang, Henan Province, China 453000; ^5^Institutes of Health Central Plains, Xinxiang Medical University, 601 Jinsui Street Xinxiang, Henan Province, China 453003

## Abstract

Gas explosion can lead to serious global public health issues. Early period gas explosion injury (GEI) can induce a series of histopathologic and specific metabolic changes. Unfortunately, it is difficult to treat GEI thoroughly. To date, the specific molecular mechanism of GEI is still unclear. To accurately diagnose and provide comprehensive clinical intervention, we performed a global analysis of metabolic alterations involved in GEI. The physiological and behavioral indicators' changes of rats after gas explosion were observed. These metabolic alterations were first investigated in a rat model using serum metabonomics techniques and multivariate statistical analysis. Significant heart rate (HR), mean blood pressure (mBP), and neurobehavioral index changes were observed in the GEI group after gas explosion. UPLC-MS revealed evident separated clustering between the control and GEI groups using supervised partial least squares discriminant analysis (PLS-DA). We designed an integrated metabonomics study for identifying reliable biomarkers of GEI using a time-course analysis of discriminating metabolites in this experiment. The metabonomics analysis showed alterations in a number of biomarkers (21 from serum). The meaningful biomarkers of GEI provide new insights into the pathophysiological changes and molecular mechanisms of GEI, including the disturbances in oxidative stress and neuroinflammatory reaction, as well as in metabolism of lipids, glucose, and amino acids in rats, suggesting that the process of GEI in humans is likely to be comprehensive and dynamic. Correlations between the GEI group and the biomarkers identified from the rat model will be further explored to elucidate the metabolic pathways responsible for GEI in the human body.

## 1. Introduction

In recent years, the number of major disasters, such as gas explosions, has been increasing worldwide [1]. Gas explosion accidents result in substantial direct and indirect economic loss in China every year, which could severely limit the development of the coal industry [2]. Gas explosion is a common accident in the coal mine industry that seriously threatens the state of the property and the safety of miners. The lack of effective use of devices makes the prevention of explosion to be greatly limited, resulting in an occasional happening of vicious gas explosion [3].

Gas explosion can generate shock wave and heat that cause injury and aggravates wounds via the interaction of blast and burns [4]. Mortality for gas explosion-related burns is significantly higher than mortality for all burn victims [5]. Our team has successfully established an animal model of gas explosion injury, which serves as a foundation for further research on the pathological mechanism of gas explosion. In the coal mine roadway, workers are directly exposed to the explosion environment due to the lack of protection for the body, which greatly increases the probability of injury caused by the blast wave. Therefore, blast wave is very likely to lead to severe damage to the whole body.

In previous animal studies, we found that gas explosion could regulate the expression of the genes of PKC and C-fos in nerve cells [6]. Gas explosion injury can activate NF-*γ*B and induced acute injury to the lung [7]. Abnormality of the corpus callosum had been found in coal mine gas explosion-related posttraumatic stress disorder [8]. Epidemiological studies have shown that the gas explosion accident in Hangzhou caused massive casualties with complex injuries [9]. In addition, coalminers with gas explosion-related posttraumatic stress disorder (PTSD) exhibited decreased hippocampal volume and structural covariance with ipsilateral amygdala [10]. A cross-sectional follow-up study showed that the quality of life continues to be affected among residents of the community after the gas explosion. Although some precautionary measures have been taken [11], most of them have limiting effects on preventing gas explosion [12]. Our current understanding of the pathology and pathophysiology of GEI is limited by the lack of in-depth and systematic research. Effective clinical treatment is absent, and the injury mechanism of GEI remains unclear. For the aforementioned reasons, a sensitive and noninvasive tool is needed to accurately assess gas explosion status and to identify biomarkers that can elucidate the mechanisms underlying GEI caused by gas explosion.

Metabonomics is a multifunctional discipline for the quantitative measurement of global, dynamic metabolic response of living systems to pathophysiological stimuli or perturbations [13]. It can be used to illuminate the dynamic responses of organisms to disease or environmental changes [14]. Ultraperformance liquid chromatography (UPLC) coupled with mass spectrometry (MS) has been used widely in metabonomics for its high chromatographic resolution, high sensitivity, and rapid separation [15]. To reduce the complexity of MS spectra, simplify data interpretation, and screen for potential biomarkers, investigators have used multivariate statistical methods, such as principal component analysis (PCA) and partial least squares discriminant analysis (PLS-DA), in metabonomics studies [16]. The focus on metabonomics research has been on variation in the content of endogenous molecules, which reflects the pathological status of living organisms and their responses under external stimulation [17]. The endogenous metabolites in the living organism respond to gas explosion, and this response can be accurately characterized by the metabonomics method. The occurrence and development mechanism of injury at the overall metabolic level can be further clarified. Identifying these markers through metabonomics is critical for early diagnosis, efficacy evaluation, and prognosis of injury related to gas explosion [18].

We designed a serum metabonomics study using gas explosion-exposed rats. LC-MS/MS combined with multivariate statistics was employed to identify valuable biomarkers and to explore the metabolic changes related to the pathogenesis of GEI. We established an animal model of GEI in a real roadway environment using a controlled explosive system and investigated the effect of gas explosion on the serum metabolic profiling of GEI rats. We also identified biomarkers, elucidated the biochemical pathways, and analyzed the possible molecular mechanisms underlying GEI. In the future, we hope to further decipher the relationships between the identified biomarkers from this study and GEI in order to explore the use of these biomarkers for clinical diagnosis and treatment of patients with GEI.

## 2. Materials and Methods

### 2.1. Chemicals and Reagents

High-performance liquid chromatography- (HPLC-) grade formic acid was purchased from American Merck company (CAS: 64-18-6, LC-MS grade). HPLC-grade ammonium formate was purchased from American Sigma company (CAS: 540-69-2, 99.0% purity). HPLC-grade methanol was purchased from China WoKai company (CAS: 67-56-1, 99.0% purity). HPLC-grade acetonitrile was purchased from American Merck company (CAS: 75-05-8, 99.0% purity). Distilled water was filtered using a Milli-Q system (Arium® mini, Sartorius). Leucine enkephalin used in HPLC was purchased from Sigma–Aldrich (St. Louis, MO, USA). Other standards used in this study were purchased from Sigma (Sigma–Aldrich, St. Louis, MO, USA, 99% purity). All other chemicals, reagents, and buffers were analytical grade products from Amresco Llc. (Solon, OH, USA).

### 2.2. Experimental Animals

Sixteen male Sprague-Dawley rats (8 weeks) with an average weight of 200 ± 20 g were purchased from the Laboratory Animal Center of the Third Affiliated Hospital of The Third Military Medical University. They were housed in a specific environment under controlled temperature (22 ± 2°C) and a humidity of 50–60%, with a 12 h light/dark cycle for one week, with free access to food and water.

After adaptation for 7 days, rats were randomly chosen for the gas explosion injury (GEI) model exposed to explosion from a distance (*n* = 8/group): control group (nonparticipation) and GEI group (40 meters away from the explosion center. Our previous study has established that this distance from explosion is reliable for developing a stable rat model of severe GEI. Before exposure, the rats were transferred to the roadway environment and placed in a fixed cage under sodium pentobarbital anesthesia. Rats were transferred to a lab quickly in a controlled time for 30 min after the gas explosion from the roadway and sacrificed under sodium pentobarbital anesthesia at the 48th hour after gas explosion. The rats' general health and behavior were observed every two hours in the lab until death, and we injected a certain amount of painkillers if the rats had obvious trauma during the survival period of 48 hours. We performed an ignition explosion before the rats waked up under the gas explosion roadway in the Chongqing Coal Academy of China, which simulates gas explosion in a coal mine. Open field tests were launched before the rats were sacrificed at the 48th hour after gas explosion. Meanwhile, the heart rate and mean arterial pressure in rats were monitored using the 2BP98A blood pressure system.

The researchers involved in this study were systematically and specifically trained before the experiment, including methods of anesthesia, placement, postinjury treatment, transfer, postinjury monitoring, and execution. The ultimate goal of all training is to alleviate the pain and unnecessary pain of laboratory animals. All animal experiments were performed in accordance with the Institute of Zoology Animal and specifically approved by the Medical Ethics Committee of Xinxiang Medical University and were in accordance with the current Chinese legislation, in addition to international standards (NIH publications No. 80-23 revised 1996).

### 2.3. Roadway Parameters and Fixtures

The roadway was 3 m in diameter and 300 m in length. According to the environment of gas explosion roadway, the volume of the mixed gas was 100 m^3^. Special gas with an oxygen concentration of 8~20% and a gas concentration of 9~9.5% was selected. The ignition energy was 20 J. The following are explosion parameters: gas capacity of 1.36 m^3^, maximum pressure value of 0.03864 MPa, maximum pressure value time of 0.699 s, a flame front velocity of 162 m/s, and a peak temperature of 853°C.

### 2.4. Open Field Tests

Neurobehavioral ability was measured in a 100 cm × 100 cm open field arena (100 cm × 100 cm × 50 cm, length × width × height, respectively). All the rats underwent a 3 min adaptation, followed by a 5 min exploration period within the testing zone. They were video-recorded using Spain Pan Lab Smart 3.0. The apparatus was cleaned with a 75% alcohol solution before the next trial. All experiments were performed under the same experimental conditions.

### 2.5. Sample Collection and Processing

Rats were transferred to a lab and sacrificed under sodium pentobarbital anesthesia at 48 h after gas explosion. Blood samples were obtained from the abdominal aorta before the rats were sacrificed and prior to blood clotting. Serum was obtained by centrifugation of the blood at 3000 rpm for 15 min in a refrigerated centrifuge and then immediately stored at -80°C. The brain of rats was extracted, and general observation was performed. The brain mass was weighed for damage assessment.

Before metabonomics analysis, the frozen serum samples were thawed at 4°C. After the samples had completely thawed, 200 *μ*L of each sample was transferred into 1.5 mL centrifuge tubes, to which 800 *μ*L of methanol (precooled at -20°C) was added. The tubes were vortexed for 60 s using MixStar (QL-866, Vortex Mixer) and centrifuged for 10 min at 12,000 rpm 4°C. The supernatant from each tube was transferred into another 1.5 mL centrifuge tube, and the samples were blow-dried by vacuum concentration. To dissolve the samples, 300 *μ*L of methanol aqueous solution (4 : 1, 4°C) was added, and the samples were filtered through a 0.22 *μ*m membrane (0.22 *μ*m PTFE, Jin Teng) in order to obtain the prepared sample extracts for LC-MS. For the quality control (QC) of the samples, a mixture containing 20 *μ*L from each prepared sample extract was prepared.

### 2.6. Quality Control Sample (QC) Preparation and Methodological Validation

Quality control samples (QCs) have been used to ensure that the results obtained from global metabolic profile study are valid. A QC typically contains pooled serum sample, prepared by mixing aliquots of the samples to be analyzed. The QC is designed to broadly represent the whole sample set. In this study, QCs were injected at regular intervals (i.e., for every ten test samples) throughout the analytical run with the goal to generate a dataset from which precision and repeatability of the method can be assessed. Meanwhile, serum standard solutions of lower (1 ng/mL), medium (20 ng/mL), and higher (400 ng/mL) concentrations were prepared as QCs by the same procedure for methodological validation purpose. All samples were stored at -20°C until the time of analysis.

Any identified deviations of the analytical results from the pooled samples were compared to the margin of errors caused by the analytical instrument. Then, 300 *μ*L acetonitrile was added to the mixture for precipitation. The sample was then subjected to vortex shaking for 2 min and centrifugation at 12000 × g for 10 min. The supernatant (100 *μ*L) obtained was added to 100 *μ*L ultrapure water. After vortexing for 30 s, 5 *μ*L of the sample was used for ultraperformance liquid chromatography/mass spectrometry (UPLC/MS) analysis.

### 2.7. Conditions of Chromatography and Mass Spectrometry

Chromatographic separation was accomplished in a Thermo Ultimate 3000 system equipped with an ACQUITY UPLC® HSS T3 (150 × 2.1 mm, 1.8 *μ*m, Waters) column maintained at 40°C. The temperature of the autosampler was 8°C. Gradient elution of analytes was carried out with 0.1% formic acid in water (A) and 0.1% formic acid in acetonitrile (B) or 5 mM ammonium formate in water (C) and acetonitrile (D), at a flow rate of 0.25 mL/min. Injection of 2 *μ*L of each sample was performed after equilibration. An increasing linear gradient of solvent B (*v*/*v*) was added as follows: 0~1 min, 2% B/D; 1~9 min, 2~50% B/D; 9~14 min, 50~98% B/D; 14~15 min, 98% B/D; 15~15.5 min, 98~2% B/D; and 15.5~17 min, 2% B/D.

The ESI-MSN experiments were executed on the Thermo Q Exactive Focus mass spectrometer with a spray voltage of 3.8 kV and -2.5 kV in positive and negative modes, respectively. Sheath gas and auxiliary gas were set at 45 and 15 arbitrary units, respectively. The capillary temperature was set at 325°C. The analyzer scanned over a mass range of *m*/*z* 81-1,000 for a full scan at a mass resolution of 70,000. Data-dependent acquisition MS/MS experiments were performed with HCD scan. The normalized collision energy was 30 eV. Dynamic exclusion was implemented to remove unnecessary information regarding the MS/MS spectra. In addition, the repeatability of the PLS-DA was evaluated using a representative pooled QC sample.

### 2.8. Metabolite Identification and Metabolic Pathway Analysis

The ProteoWizard software (V3.0.8789) was used to convert the obtained original data into mzXML format (XCMS Input file Format). R (v3.3.2) XCMS package was employed for peak identification, peak filtration, and peak alignment, and the main parameters are bw = 5, ppm = 15, peakwidth = c (10, 20), mzwid = 0.015, mzdiff = 0.01, and the method = centWave. The data matrix including mass to charge ratio (M/Z), retention time (RT), and intensity was obtained. In the positive ion mode, 9,806 precursor molecules and the negative ion mode, 8,322 precursor molecules were obtained, and the data were exported to Excel for subsequent analysis. The batch normalization of the data peak area was performed to compare data of different orders of magnitude.

All data were introduced into R language ropls package for multivariate statistical analysis via principal component analysis (PCA). For biomarker identification, the ions, which were mostly responsible for the variances, were identified based on variable importance in the projection (VIP) and exact masses. The metabolites with VIP values > 1.0 in the model were regarded as potential biomarkers. R language ropls package was used to perform powerful multivariate analysis in principal component analysis (PCA), partial least squares discriminant analysis (PLS-DA), and orthogonal projection to latent structure discriminate analysis (OPLS-DA).

The probable formulas for the potential biomarkers were first identified according to accurate mass measurement (mass error < 20 ppm) and by considering the relative intensities of the isotope peaks based on high-resolution MS spectra. Then, the MSMS mode was used to facilitate the fragment ion-analysis process using chemically intelligent peak-matching algorithms. Briefly, the UPLC/MS/MS product ion spectra of potential biomarkers were matched with the structural data of metabolites acquired from the Human Metabolome Database (HMDB) (http://www.hmdb.ca), Metlin (http://metlin.scripps.edu), massbank (http://www.massbank.jp/), Lipid Maps (http://www.lipidmaps.org), mzCloud (https://www.mzcloud.org), and BioNovoGene Company (http://www.bionovogene.com) standard database. The implicated pathways associated with the biomarkers were described using databases Kyoto Encyclopedia of Genes and Genomes (KEGG, http://www.genome.jp/kegg/). In addition, other implicated pathways of biomarkers were further interpreted based on previous literatures.

### 2.9. Data Processing and Analysis

Data were collected before transferring into R language ropls package for multivariate statistical analysis. After identification and alignment, low-molecular-weight metabolites were presented as chromatographic peaks at the base peak intensity (BPI) chromatograms. Through multivariable statistical analysis, the resultant 3D matrix containing arbitrarily assigned peak indices (retention time-m/z pairs), sample names (observations), and normalized ion intensities for each peak area were exported to R language ropls package. Using R language ropls package, the score plot was visualized and the greatest variable importance in projection (VIP) values was determined by partial least squares discriminant analysis (PLS-DA). Pareto scaling was used prior to multivariate statistical analysis to avoid chemical noise.

Student's *t*-test was used for statistical analysis with SPSS (version 17.0; Beijing Stats Data Mining Co. Ltd, Beijing, China). All *P* values were subjected to a 2-tailed test, and a *P* value < 0.05 was considered significant for all statistical analyses in this study. The false discovery rate (FDR) calculation was performed. Data were presented as the mean plus or minus the standard deviation (SD).

## 3. Results

### 3.1. Changes in Brain Tissue Damage

**Figure 1:**
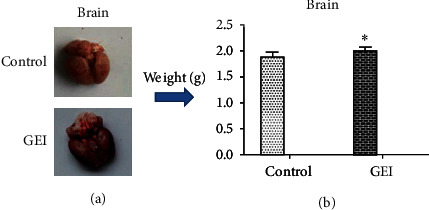
Changes in brain appearance (a) and weight (b) after gas explosion. Control: control group; GEI: gas explosion injury group. Data are expressed as the mean ± SD (*n* = 8). ^∗^*P* < 0.05, significantly different from the control.

In this study, the injuries observed in rats exposed to gas exploration were GEI. Compared with the normal control group, gas explosion exhibited an obvious effect on the brain of rats in the GEI group, as reflected by obvious cerebral congestion and increased weight of brain tissue (Figure 1). In addition, different degrees of GEI also occurred in other parts of the body, indicating that the rat model of GEI was established successfully.

### 3.2. Open Field Tests

**Figure 2:**
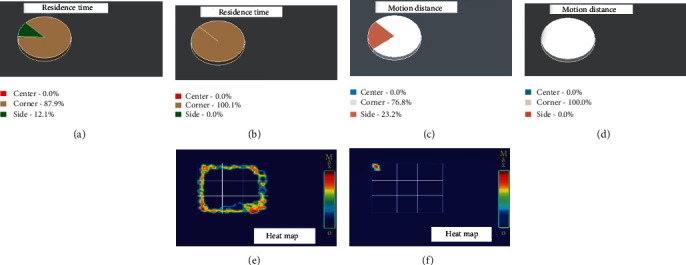
Residence time (a, b), motion distance (c, d), and trajectory heat map (e, f) of rats in various regions from the open field test. (a, c, e) Control group and (b, d, f) gas explosion injury group.

Abnormalities in the motor function of rats, such as apnea, slow heart rate, decreased mean arterial pressure, and other brainstem inhibition phenomena (shown in S1 Table), were also detected after gas explosion. These characteristic physiological changes were caused by the direct effects of the blast wave on the rats' skull. We also observed decreased motion distance and trajectory of rats, while residence time was increased in the GEI group in an open-field experiment, indicating that gas explosion could result in brain nerve and impaired the neurological behavioral function in rats (Figure 2).

### 3.3. Methodological Validation

The precision and repeatability of the LC-MS method were validated by the reduplicate analysis of six injections of the same QCs and six parallel samples prepared using the same preparation protocol, respectively. During the process of sample extraction and test analysis, differences could be observed among the QC samples. Generally, the smaller the difference, the higher the stability of the method and the better quality of the data. We observed a dense distribution of the QC samples, which is presented in the diagram of principal component analysis (PCA) score plots. The clustered and reproducible QC samples are shown in S2 Figure. A and C indicate that the system is stable. For the purpose of quality control (QC), quality assurance (QA) is usually carried out to remove the characteristic peak of the poor repetitive features in the QC sample in order to obtain a higher quality data set. In this study, the proportion of characteristic peaks of QC samples with RSD < 30% could reach about 70%, which suggested that the precision and repeatability of the proposed method were acceptable for metabonomics analysis (S2 Figure B and D).

### 3.4. UPLC-MS/MS Fingerprinting and Multivariate Analysis

**Figure 3:**
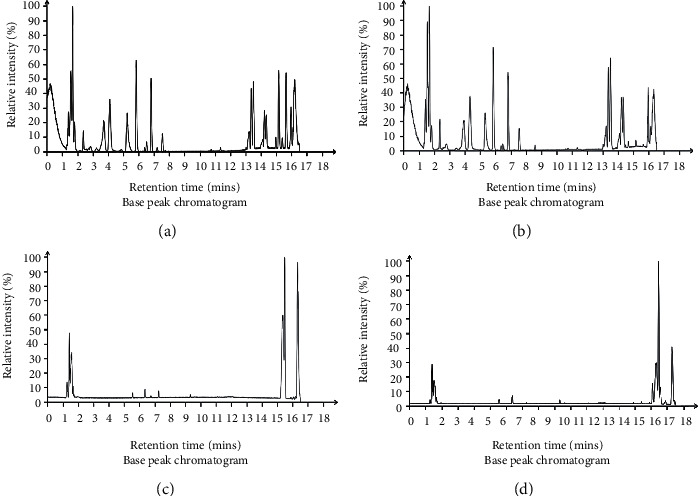
Representative positive (a, b) and negative (c, d) BPI chromatograms of serum obtained from the control (a, c) and GEI (b, d) groups. BPI: base peak intensity; control: control group; GEI: gas explosion injury group.

In this study, all rat serum samples collected were analyzed in both positive and negative ionization modes for serum metabolic profiling, in order to cover as many compounds as possible. Representative positive and negative BPI chromatograms of serum obtained from the control and GEI groups are shown in Figure 3.

**Figure 4:**
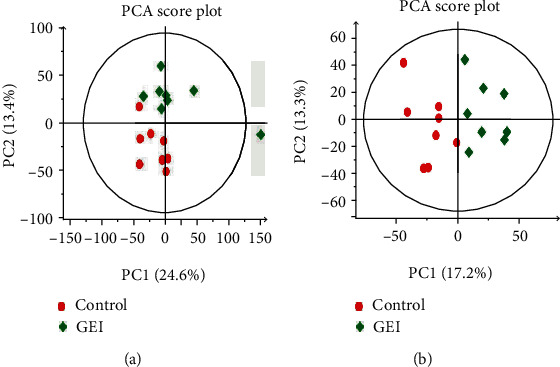
PCA score plot derived from the UPLC-MS/MS of serum obtained from the control and GEI groups. (a) Positive ion mode. (b) Negative ion mode. Control: control group (red round); GEI: gas explosion injury group (diamond); PCA: principal component analysis. Each data point represents one subject.

After applying UPLC-MS in both positive ionization modes, some significant metabolic alterations between the control and GEI groups could be observed visually. In addition, unsupervised PCA and supervised PLS-DA and OPLS-DA models were constructed based on the two groups to understand the subtle metabolic changes and to characterize the metabolite profile of the control and GEI samples. PCA was performed to identify the changes in metabolite profiles on positive ESI data. As shown in the PCA score plot in Figure 4, the data plots of the three treated groups were separated from those of the control group, although some overlaps still existed between the treated and the control groups.

**Figure 5:**
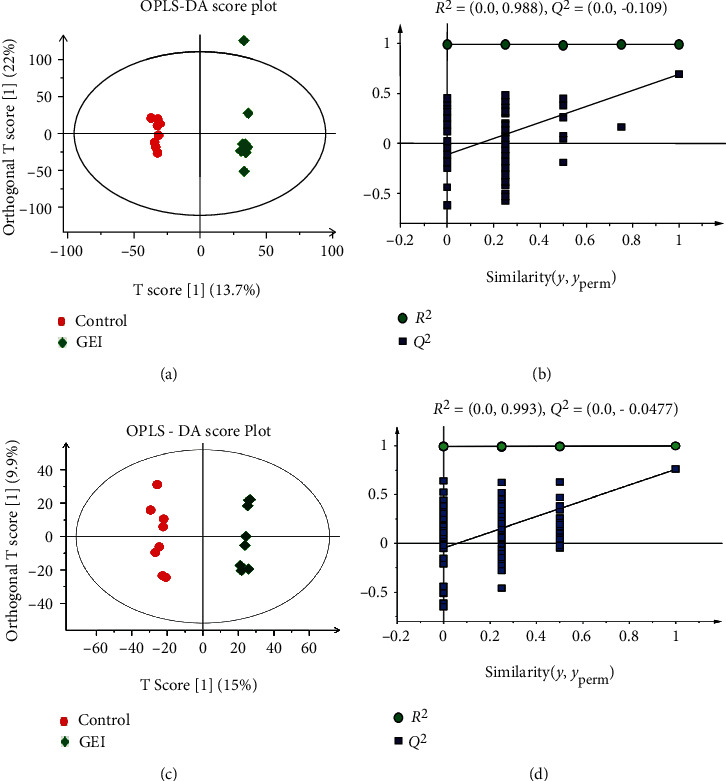
OPLS-DA score plot and permutation test for OPLS-DA derived from the UPLC-MS/MS of serum obtained from the control and GEI groups. (a, b) Positive ion mode and (c, d) negative ion mode. Control: control group (red round); GEI: gas explosion injury group (diamond); OPLS-DA: orthogonal projection to latent structure discriminant analysis. Each data point represents one subject. The *R*^2^*Y* value represents the goodness of fit of the model, and the *Q*^2^ value represents the predictability of the models.

PLS-DA is a multivariate metabonomics data analysis method based on PLS, and PLS-DA analysis maximizes the product of the variance matrix of measured variables and the correlation of measured data with properties of interest. The resulting score plot of PLS-DA and OPLS-DA (*R*^2^*Y* = 0.99 and *Q*^2^ = 0.55, S3 Figure A and B; *R*^2^*Y* = 0.988 and *Q*^2^ = −0.109, Figures 5(a) and 5(b)) in the positive mode and negative mode (*R*^2^*Y* = 0.96 and *Q*^2^ = −0.2, S3 Figure C and D; *R*^2^*Y* = 0.993 and *Q*^2^ = −0.0477, Figures 5(c) and 5(d)) for the GEI, and the control groups gradually formed two separate clusters in the PLS-DA and OPLS-DA plots at the 48th hour after gas explosion. To evaluate the possibility of error that the present PLS-DA and OPLS-DA models might generate, one hundred times of the permutation test for PLS-DA and OPLS-DA were applied. All *R*^2^*Y* and *Q*^2^ values to the left were lower than the original points to the right (S3 Figure and Figure 5), showing that the PLS-DA and OPLS-DA models were valid. These results suggested that GEI could lead to serum metabolite alterations in the rat model established in our experiment.

### 3.5. Potential Biomarkers

Potential biomarkers were identified based on the variable importance in the projection (VIP) parameters of loading plots. To elucidate the elemental composition of each biomarker, twenty-one biomarkers were confirmed by comparing their retention times and MS/MS fragmentation patterns using LC-MS/MS. They were also identified by searching several free online databases. Accordingly, nine ions in the serum from the positive mode were initially identified, while twelve ions in the serum from the negative mode were initially identified. All biomarkers were determined based on exact mass, isotopic distribution, and mass spectra fragmentation patterns using MassFragment software. The *m*/*z* and retention time, postulated identity, elemental composition structural formula, mass fragment information, RSD, and QC data of identified biomarkers in this experiment are shown in Tables 1 and 2, S4 Table, and S5 Table. The identification figures, chemical structures, box figures, and bar figures of these metabolites identified in rat serum in the control and GEI groups at the 48^th^ hour after gas explosion are shown in S6 Figure, S7 Figure, S8 Figure, and S9 Figure, respectively. Compared with the control group, a significant decrease in the intensity of citrulline, L-glutamine, and glycochenodeoxycholic acid was observed, whereas L-lysine, L-methionine, urocanic acid, 2-aminoadipic acid, L-threonine, and L-phenylalanine were significantly increased in the positive ion mode in the GEI group at 48 hours after gas explosion (*P* < 0.05). A significant decrease in the intensity of itaconic acid, aconitic acid, citric acid, galactitol, indoleacrylic acid, indoxylsulfuric acid, and xanthurenic was observed, whereas L-glutamate, L-aspartic acid, cholic acid, estrone 3-glucuronide, and taurine were significantly increased in the negative ion mode in the GEI group at 48 hours after gas explosion (*P* < 0.05).

## 4. Discussion

Gas explosion can lead to unique combined injuries via complicated mechanism. In order to establish the rats' model of gas explosion injury (GEI), various factors and mechanisms of GEI can be summarized from the experimental technical parameters, which provided a technical basis for future researches on related topics. A primary goal of previous studies has been to develop treatments that would lower the extent of damage associated with GEI. However, none of these studies has been able to elucidate the pathogenic mechanisms of GEI. Therefore, noninvasive methods, along with sensitive and specific biomarkers, are urgently needed for the diagnosis and progression monitoring of GEI. Serum metabonomics based on UPLC-MS/MS has been of great value in the discovery of biomarkers and the elucidation of the pathogenesis of various diseases [19]; therefore, we hypothesized that a similar method could be used to evaluate GEI. To date, there has been no report on serum metabonomics that focused on GEI. The current serum metabonomics study based on UPLC-MS/MS, coupled with multivariate statistical analysis, is the first of its kind for the identification of potential biomarkers and clarification of the molecular mechanisms of GEI.

Our results showed that the required explosion energy could be predicted by controlling the volume of gas. Furthermore, our data demonstrated that the explosion parameters evaluated in this study were accurate and reliable and exhibited good repeatability and stability. The animal model used was also reproducible and stable. After the explosion experiment, rats in the GEI group suffered GEI in parts of the body to varying degrees, suggesting that an animal model of GEI was successfully established. The increase in brain weight and the hemorrhage observed from the GEI provided further support the validity of the animal model (Figure 1). PCA is an unsupervised pattern recognition method initially used to discern the presence of inherent similarities in spectral profiles. PCA can effectively demonstrate the differences between the exposure and control groups. So an unsupervised PCA model was built to further characterize the serum metabolite profile of the exposed animals and examine any systemic metabolic changes. As shown in Figure 4, the result of the PCA model of serum from the GEI and control groups and the GEI group gradually formed a completely separated cluster from the control group after the explosion, indicating that gas explosion remarkably disturbed the normal serum metabolic profiles of rats in the exposed group. A complete segregation between the control and GEI groups was observed on the score plot of the PCA and OPLS-DA models (Figures 4 and 5). Various types of validation methods also demonstrated that the model was effective and reliable.

**Figure 6:**
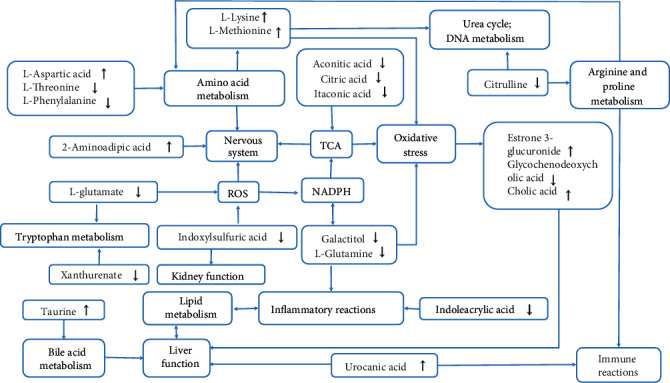
The altered pathways and injury effects in response to gas explosion. The upward or downward arrows represent an elevated or decreased level of metabolites in the GEI group compared with the control group. GEI: gas explosion injury.

Twenty-one metabolites were identified as significantly altered in the GEI group when compared with the control group, and ten were downregulated, while eleven were upregulated (Tables 1 and 2). Furthermore, the *Z*-score of biomarkers identified from the control and GEI groups is shown in S10 Figure. We hypothesized that these metabolites might represent potential markers for GEI. In addition, the biological function of the individual biomarker could provide important clues to the pathophysiologic mechanism of GEI. Based on the analysis of the KEGG pathway, some important metabolic pathways were disturbed in GEI rats (Figure 6 and S11 Figure).

The first pathway is involved in energy metabolism, inflammation responses, bile acid metabolism, and lipid metabolism. Both aconitic acid and citric acids are closely related to glyoxylate and dicarboxylate metabolism. Itaconic acid is derived from succinic acid. It is an intermediate in the C5-branched dibasic acid metabolism and a substrate for the enzyme succinate-CoA ligase (ADP-forming) [18]. The decreased level of itaconic acid in our study indicated that gas explosion could affect the Kreb's cycle in rats, which further suggested that GEI is closely related to energy metabolism. These results shed light on future development of treatments that aim to reduce the incidence of GEI. Additional studies are needed to further reveal the relationship between GEI and energy metabolism. A study has recently reported that indoleacrylic acid (IA) can mitigate inflammatory responses [20]. Therefore, stimulating indoleacrylic acid production could promote anti-inflammatory responses with potential therapeutic benefits [21]. The results from this study implied that rapid expenditure of energy could lead to the modification of the metabolic pathway in order to provide a sufficient amount of energy for bodily functions. In addition, the body also reacts to gas explosion by avoiding oxidative damage and chronic inflammation, which may help reduce the severity of GEI. Chronic inflammation is an important factor of GEI [22] and could influence rat lipid metabolism [23]. The metabolism of cholesterol and phospholipids is a primary function of a normal rat liver, and downregulated synthesis of bile acids may lead to the malabsorption of lipids. The significant increase in taurine and decrease in glycine-conjugated bile compound in rat serum suggested that the cometabolism of gut microbes was altered in rats, and potential changes in the enterohepatic circulation might have also occurred. Moreover, bile acids are closely correlated with lipids, which could have potential regulatory effects on nuclear receptors [24]. The abnormal metabolism of phospholipids can also affect many biological processes, such as inflammation [25].

The second pathway is involved in galactose, glutamate, and glutathione metabolism and antioxidant system. Excess lactose consumption in individuals with galactose intolerance or galactosemia could activate aldose reductase, which produces galactitol, and thereby depletes NADPH and leads to decreased glutathione reductase activity [26]. In hemodialyzed patients, serum levels of indoxyl sulfate are associated with levels of pentosidine, a marker for carbonyl and oxidative stress. In vitro, indoxyl sulfate increases reactive oxygen species (ROS) production of tubular cells and promotes NADPH oxidase activity in endothelial cells, which may further protect the brain by maintaining glutathione reductase activity. Glutamine is a major nitrogen carrier and a carbon substrate for anabolic processes in cancer cells, and it may protect rats from oxidative stress [27]. Similarly, glutathione, which is the major redox couple in animal cells [28], was also increased. The increase in glutathione reflected the alteration of the redox state, which is one of the key performance indicators in various pathologic conditions, especially in traumatic and inflammatory injuries [29]. 2-Aminohexanoic acid, a metabolite in the principal biochemical pathway of lysine, antagonizes neuroexcitatory activity modulated by the glutamate receptor. 2-Aminoadipate is also known to be a potential small-molecule marker of oxidative stress [30]. Increased 2-aminoadipic acid levels in serum indicate disruption in lysine metabolism. Previous research has demonstrated that L-glutamate is a nonessential amino acid that is naturally present in the L-form. Glutamic acid is the most common excitatory neurotransmitter in the nervous system. Increased 2-aminoadipic acid in the positive ESI modes and increased L-glutamate in the negative ESI modes in our experimental indicated that GEI could induce disruption of the central nervous system through oxidative stress and reactive oxygen species (ROS). Glutamine is an important energy source for many cells. In glutamic acid-to-glutamine conversion, an ammonia group is added to glutamic acid, a reaction that is catalyzed by glutamine synthase. This agent is a substrate for the production of both excitatory and inhibitory neurotransmitters (glutamate and GABA) and is an important source of energy for the nervous system. Decreased levels of L-glutamine in rat serum after gas explosion suggested that the nervous system is in a state of energy deficiency, which further indicated that GEI can refrain the nervous system from its normal function. Open field test (Figure 2) provided further support to these findings.

The third pathway is involved in oxidative stress, DNA damage, and the nervous system. The origin of many diseases is closely related to the oxidative damage caused by free radicals. In this study, GEI was, to a certain extent, also associated with oxidative stress induced by gas explosion. The mechanism for GEI induced by glycochenodeoxycholic acid, cholic acid, estrone 3-glucuronide, and taurine may involve the generation of oxidative stress. Oxidative damage is the primary driver for GEI; therefore, an elevated level of antioxidative metabolites was expected in GEI rats. In fact, decreased levels of some metabolites that could protect GEI rats from oxidative damage were observed. The substantial decline in galactitol suggested a decreased flux of metabolic fuels to support galactose metabolism, which is crucial for the production of NADPH that could modulate the perceived increase in oxidative stress and neuroinflammation in GEI [31]. Aspartic acid, a nonessential amino acid in humans, is made from glutamic acid by enzymes using vitamin B6. Aspartic acid plays important roles in the urea cycles, DNA metabolism, and the citric acid cycle. Lysine, methionine, and some nucleotides are synthesized from aspartic acid. Elevated levels of L-aspartic acid in the GEI group suggested a need to synthesize more L-lysine and L-methionine to satisfy the demands of the urea cycle and DNA metabolism. As important amino acids of the nervous system, phenylalanine and threonine play crucial roles in fat metabolism and have been used to relieve anxiety and mild depression. The increased levels of L-phenylalanine and L-threonine in this study indicated that neurological dysfunction could be attributed to GEI. Open field test conducted in this study (Figure 2) provided further evidence that the nervous system was affected by gas explosion. Notably, rats exposed to gas explosion exhibited an increase in the extent of tension, such as increased residence time and decreased motion distance in a quiet environment.

The fourth pathway concerns the liver, kidney, and immune system dysfunction. Cholic acid and glycochenodeoxycholic acid are major primary bile acids produced in the liver and usually conjugated with glycine or taurine. They can facilitate fat absorption and cholesterol excretion. The decreased level of glycochenodeoxycholic acid in our study also indicated that gas explosion could affect the fat absorption and cholesterol excretion in rats through inhibition of liver function. Urocanic acid (UA) is a deamination product of histidine breakdown. In the liver, UA is an intermediate in the conversion of histidine to glutamic acid and potentially serving as an immunoregulator. Increased levels of UA in the GEI group indicated that gas explosion affected the glutamic acid levels in rats through histidine metabolism. Therefore, GEI could induce immune reactions [32]. Indoxylsulfuric acid is a circulating uremic toxin that stimulates glomerular sclerosis and interstitial fibrosis, which increase the rate of progression of renal failure. Citrulline is produced in the urea cycle or as a by-product of arginine in the production of NO. An important metabolic function of arginine is to promote wound healing, by promoting the synthesis of collagen tissue that repairs wounds. The immunomodulatory function of arginine can prevent the degeneration of thymus (especially after an injury), and the supplement of arginine can increase the weight of thymus and promote the growth of lymphocytes in the thymus. Therefore, the decrease in citrulline in our experimental indicated that gas explosion could decrease arginine and proline metabolism and thereby lead to immunodeficiency.

## 5. Conclusion

In summary, the metabolic profiling analysis of rat serum provided a holistic view of the metabolic features of GEI. The differential metabolites in the rat serum were filtered and identified, and the results showed that metabolism in rat was modified to promote GEI repair or suppress inflammation. The rapid consumption of energy by rats downregulated the galactose metabolism and the TCA cycle, both of which are consistent with the Warburg effect. Based on the correlation network, modified metabolism of lipid is the most important feature of the neuroinflammatory response, which may be important in protecting the body from oxidative damage. This protective effect included increased levels of antioxidative metabolites and decreased levels of inflammation-related metabolites, which are likely related to GEI. The associations between GEI and the identified biomarkers need to be further investigated in order to elucidate their potential application to human subjects. Despite certain limitations to this study, our findings could potentially bring new hope to the treatment of GEI.

## Figures and Tables

**Table 1:** Potential biomarkers identified in positive ion mode in rat serum.

Retention time (min)	Measured *m*/*z* ion (Da)	Calculated *m*/*z* ion (Da)	Exact mass	Mass error (PPM)	Elemental composition	Postulated identity	Scan mode	VIP values	KEGG	Trend in exposure groups	RSD
1.56	176.1030	176.1030	175.1858	0	C_6_H_13_N_3_O_3_	Citrulline^a,b^	[M+H]+	2.0919	C00327	↓	0.03%
1.34	147.1128	147.1128	146.1876	1	C_6_H_14_N_2_O_2_	L-Lysine^a,b^	[M+H]+	2.0666	C00047	↑	0.02%
2.35	150.0584	150.0576	149.2123	5	C_5_H_11_NO_2_S	L-Methionine^a,b^	[M+H]+	1.9141	C00073	↑	0.05%
2.34	139.0502	139.0495	138.1241	5	C_6_H_6_N_2_O_2_	Urocanic acid^a,b^	[M+H]+	1.7809	C00785	↑	0.07%
1.70	162.0761	162.0756	161.1559	3	C_6_H_11_NO_4_	2-Aminoadipic acid^a,b^	[M+H]+	1.7341	C00956	↑	0.03%
1.53	120.0657	120.0653	119.1192	4	C_4_H_9_NO_3_	L-Threonine^a,b^	[M+H]+	1.6720	C00188	↑	0.02%
5.83	166.0863	166.0860	165.1892	3	C_9_H_11_NO_2_	L-Phenylalanine^a,b^	[M+H]+	1.6682	C00079	↑	0.02%
1.52	147.0764	147.0765	146.1446	0	C_5_H_10_N_2_O_3_	L-Glutamine^a,b^	[M+H]+	1.6528	C00064	↓	0.03%
13.61	450.3212	450.3200	449.6234	3	C_26_H_43_NO_5_	Glycochenodeoxycholic acid^a,b^	[M+H]+	1.4590	C05466	↓	0.04%

^a^Ions identified by comparison to the standard chemicals. ^b^Biomarkers identified by the Human Metabolome Database (HMDB) and confirmed using exact mass data and MS fragmentation. VIP: variable importance in projection; RSD: relative standard deviation.

**Table 2:** Potential biomarkers identified in negative ion mode in rat serum.

Retention time (min)	Measured *m*/*z* ion (Da)	Calculated *m*/*z* ion (Da)	Exact mass	Mass error (PPM)	Elemental composition	Postulated identity	Scan mode	VIP values	KEGG/HMDB	Trend in exposure groups	RSD
1.29	173.0084	173.0092	174.1082	5	C_6_H_6_O_6_	Aconitic acid^a,b^	[M-H]-	2.0521	C00417	↓	0.01%
1.39	146.0449	146.0459	147.1293	7	C_5_H_9_NO_4_	L-Glutamate^a,b^	[M-H]-	1.8388	C00025	↑	0.06%
1.31	191.0191	191.0199	192.1235	4	C_6_H_8_O_7_	Citric acid^a,b^	[M-H]-	1.8020	C00158	↓	0.03%
1.39	132.0293	132.0309	133.1027	14	C_4_H_7_NO_4_	L-Aspartic acid^a,b^	[M-H]-	1.6037	C00049	↑	0.06%
10.13	407.2804	407.2803	408.5714	0	C_24_H_40_O_5_	Cholic acid^a,b^	[M-H]-	1.5278	C00695	↑	0.04%
1.53	180.9724	180.9731	182.1718	4	C_6_H_14_O_6_	Galactitol^a,b^	[M-H]-	1.4248	C01697	↓	0.01%
8.00	445.1903	445.1868	446.4910	8	C_24_H_30_O_8_	Estrone 3-glucuronide^a,b^	[M-H]-	1.3901	C11133	↑	0.06%
1.50	124.0064	124.0074	125.1480	8	C_2_H_7_NO_3_S	Taurine^a,b^	[M-H]-	1.2990	C00245	↑	0.02%
8.05	186.0554	186.0561	187.1950	4	C_11_H_9_NO_2_	Indoleacrylic acid^a,b^	[M-H]-	1.3879	HMDB00734	↓	0.04%
7.26	212.0018	212.0023	213.2100	2	C_8_H_7_NO_4_S	Indoxylsulfuric acid^a,b^	[M-H]-	1.7347	HMDB00682	↓	0.05%
6.42	203.9966	203.9985	205.0375	16	C_10_H_7_NO_4_	Xanthurenate^a,b^	[M-H]-	1.6725	C02470	↓	0.06%
1.29	129.0183	130.0193	130.099	7	C_5_H_6_O_4_	Itaconic acid^a,b^	[M-H]-	1.9791	C00490	↓	0.02%

^a^Ions identified by comparison to the standard chemicals. ^b^Biomarkers identified by the Human Metabolome Database (HMDB) and confirmed using exact mass data and MS fragmentation. VIP: variable importance in projection; RSD: relative standard deviation.

## Data Availability

The data used to support the findings of this study are included within the article.

## Supplementary Materials

Supplementary Materials S1 Table: the HR and mBP determined in rats between the control and GEI groups at the 48th hour after gas explosion. S2 Figure: two-dimensional principal component analysis (PCA) score plots of serum samples (green circle) and quality control (QC) samples (red circle) and QA results in positive and negative ion modes at the 48th hour after gas explosion. QC: quality control (A and C); QA: quality assurance; RSD: relative standard deviation. S3 Figure: PLS-DA score plot and permutation test for PLS-DA derived from the UPLC-MS/MS of serum obtained from the control and GEI groups. A, B: positive ion mode; C, D: negative ion mode; control: control group (red round); GEI: gas explosion injury group (diamond); PLS-DA: partial least squares discriminant analysis. Each data point represents one subject. The R2Y value represents the goodness of fit of the model, and the Q2 value represents the predictability of the models. S4 Table: the MS fragment ions and QC data for the potential biomarkers in positive ion mode. S5 Table: the MS fragment ions and QC data for the potential biomarkers in negative ion mode. S6 Figure: metabolites identified in rat serum in positive and negative ion modes from the control and GEI groups at the 48th hour after gas explosion. Control: control group; GEI: gas explosion injury group. S7 Figure: chemical structures of metabolites identified in rat serum in positive and negative ion modes from the control and GEI groups at the 48th hour after gas explosion. Control: control group; GEI: gas explosion injury group. S8 Figure: box figures of the metabolites identified in rat serum in positive and negative ion modes from the control and GEI groups at the 48th hour after gas explosion. Control: control group (red); S40: gas explosion injury group (green). Significantly different from the control: ^∗^*P* < 0.05; ^∗∗^*P* < 0.01, and ^∗∗∗^*P* < 0.001. S9 Figure: bar figures of the metabolites identified in rat serum in positive and negative ion modes from the control and GEI groups at the 48th hour after gas explosion. BTBI: blast-related traumatic brain injury group; control: control group (red); S40: gas explosion injury group (green). Significantly different from the control: ^∗^*P* < 0.05, ^∗∗^*P* < 0.01, and ^∗∗∗^*P* < 0.001. S10 Figure: *Z*-score of biomarkers identified in rat serum in positive and negative ion modes from the control and GEI groups at the 48th hour after gas explosion. BTBI: blast-related traumatic brain injury; control: control group (red round); S40: gas explosion injury group (green diamond). S11 Figure: pathway impacted according to the identified biomarkers derived in rat serum in positive and negative ion modes from the control and GEI groups at the 48th hour after gas explosion. GEI: gas explosion injury group.
