# Supplementary material for: Evaluation of Gas Explosion Injury Based on Analysis of Rat Serum Profile by Ultra-Performance Liquid Chromatography/Mass Spectrometry-Based Metabonomics Techniques
Source: Biomed Res Int. 2020 Jul 28;2020:8645869. doi: 10.1155/2020/8645869 (PMC7407032; doi:10.1155/2020/8645869)
Supplement: Supplementary Materials — S1 Table: the HR and mBP determined in rats between the control and GEI groups at the 48th hour after gas explosion. S2 Figure: two-dimensional principal component analysis (PCA) score plots of serum samples (green circle) and quality control (QC) samples (red circle) and QA results in positive and negative ion modes at the 48th hour after gas explosion. QC: quality control (A and C); QA: quality assurance; RSD: relative standard deviation. S3 Figure: PLS-DA score plot and permutation test for PLS-DA derived from the UPLC-MS/MS of serum obtained from the control and GEI groups. A, B: positive ion mode; C, D: negative ion mode; control: control group (red round); GEI: gas explosion injury group (diamond); PLS-DA: partial least squares discriminant analysis. Each data point represents one subject. The R2Y value represents the goodness of fit of the model, and the Q2 value represents the predictability of the models. S4 Table: the MS fragment ions and QC data for the potential biomarkers in positive ion mode. S5 Table: the MS fragment ions and QC data for the potential biomarkers in negative ion mode. S6 Figure: metabolites identified in rat serum in positive and negative ion modes from the control and GEI groups at the 48th hour after gas explosion. Control: control group; GEI: gas explosion injury group. S7 Figure: chemical structures of metabolites identified in rat serum in positive and negative ion modes from the control and GEI groups at the 48th hour after gas explosion. Control: control group; GEI: gas explosion injury group. S8 Figure: box figures of the metabolites identified in rat serum in positive and negative ion modes from the control and GEI groups at the 48th hour after gas explosion. Control: control group (red); S40: gas explosion injury group (green). Significantly different from the control: ∗P < 0.05; ∗∗P < 0.01, and ∗∗∗P < 0.001. S9 Figure: bar figures of the metabolites identified in rat serum in positive and negative ion modes from the control [file 8645869.f1.zip › 8645869.f1/Supplementary file 4.docx]

**In negative ion mode：**

**
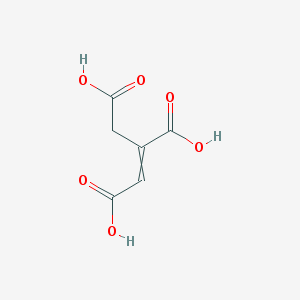
** **Aconitic acid**

**
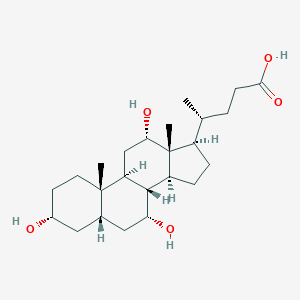
** **Cholic acid**

**
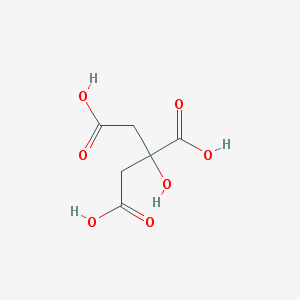
** **Citric acid**

**
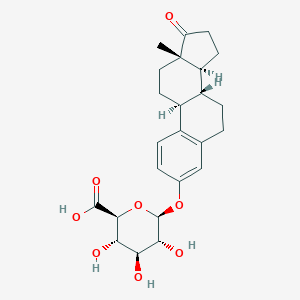
** **Estrone 3-glucuronide**

**
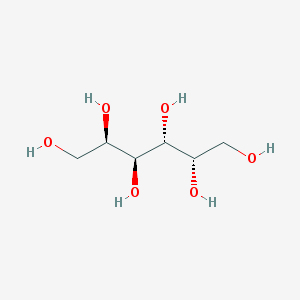
** **Galactitol**

**
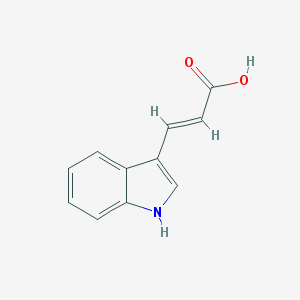
** **Indoleacrylic acid**

**
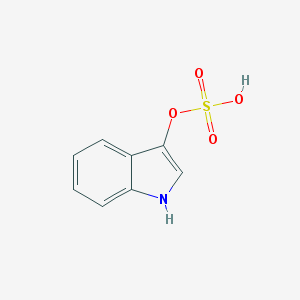
** **Indoxylsulfuric acid**

**
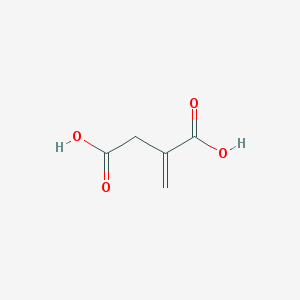
** **Itaconic acid**

**
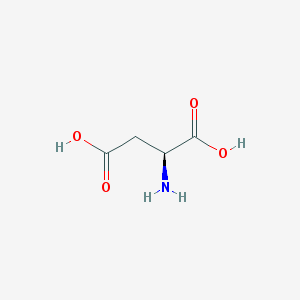
** **L-Aspartic acid**

**
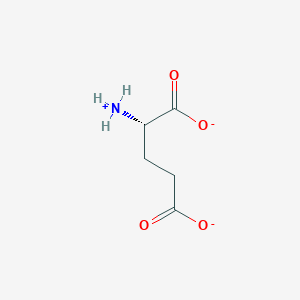
** **L-Glutamate**

**
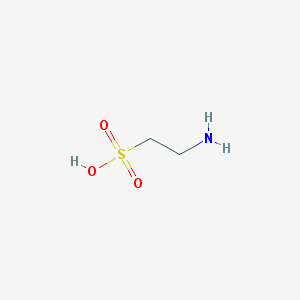
** **Taurine**

**
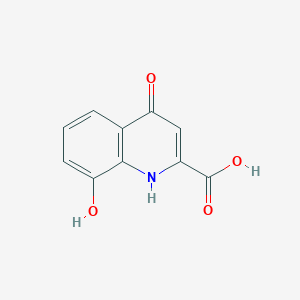
** **Xanthurenate**

**In positive ion mode：**


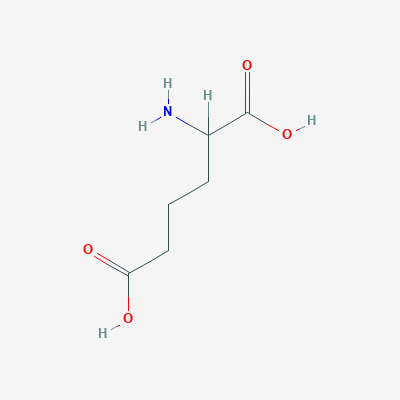
 **2-Aminoadipic acid**


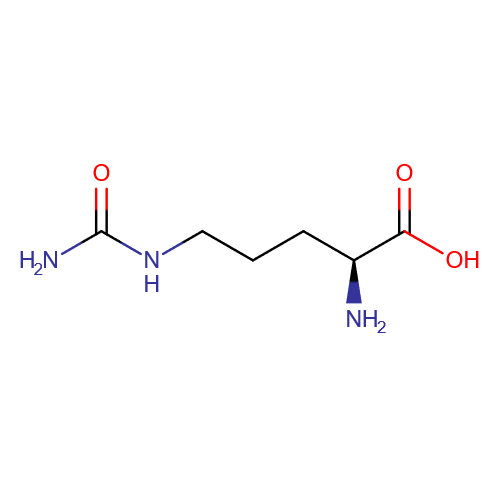
 **Citrulline** **
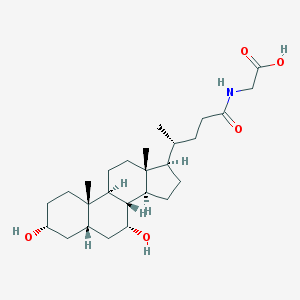
Glycochenodeoxycholic acid**

**
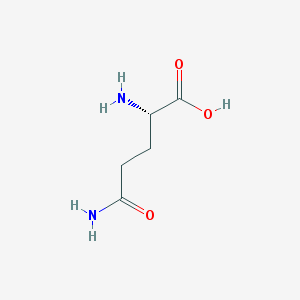
** **L-Glutamine**

**
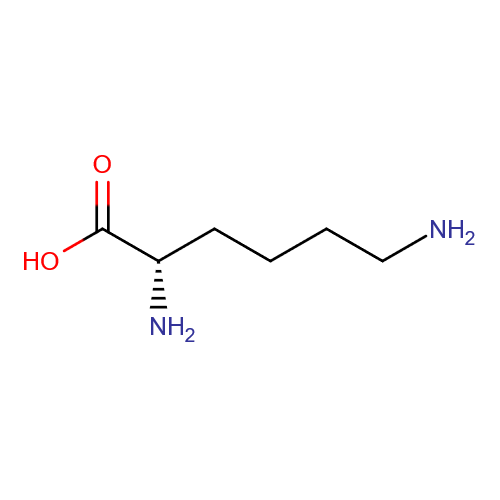
** **L-Lysine**

**
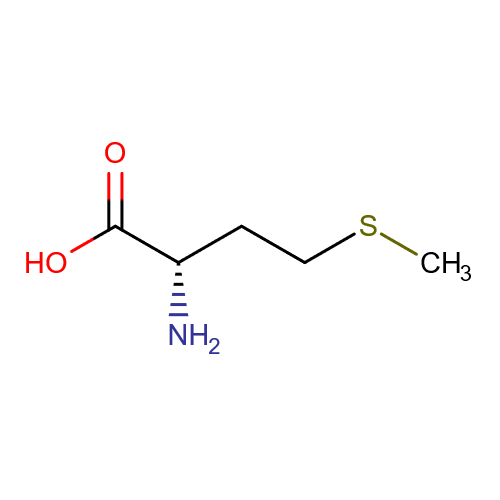
** **L-Methionine**

**
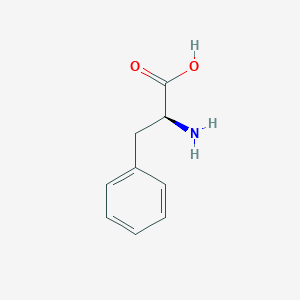
** **L-Phenylalanine**

**
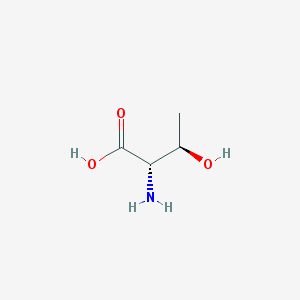
** **L-Threonine**

**
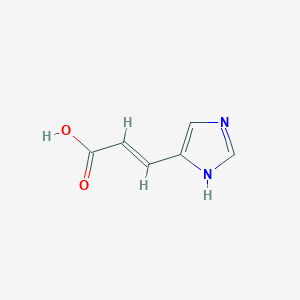
** **Urocanic acid**
